# Mammaglobin 1 mediates progression of trastuzumab‐resistant breast cancer cells through regulation of cyclins and NF‐κB

**DOI:** 10.1002/2211-5463.13468

**Published:** 2022-08-09

**Authors:** Ratih Kusumastuti, Yuji Kumagai, Seiichiro Ishihara, Atsushi Enomoto, Takashi Murakami, Motoaki Yasuda, Hisashi Haga

**Affiliations:** ^1^ Division of Life Science, Graduate School of Life Science Hokkaido University Sapporo Japan; ^2^ Department of Advanced Transdisciplinary Sciences, Faculty of Advanced Life Science Hokkaido University Sapporo Japan; ^3^ Department of Pathology Nagoya University Graduate School of Medicine Nagoya Japan; ^4^ Faculty of Medicine Saitama Medical University Moroyama Japan; ^5^ Department of Oral Pathobiological Science, Graduate School of Dental Medicine Hokkaido University Sapporo Japan

**Keywords:** cyclin, invasion, MGB1, migration, resistance, trastuzumab

## Abstract

Overexpression of human epidermal growth factor receptor 2 (HER2) in various cancers is correlated with poor patient survival. Trastuzumab, a recombinant humanized monoclonal antibody against HER2, has been considered to be a first‐line therapy for HER2‐positive breast cancer patients, but its usefulness is limited by the development of resistance. In this study, we established resistant cells by long‐term treatment with trastuzumab. These cells showed higher proliferation, invasion, and migration abilities than the wild‐type cells. Mammaglobin 1 (MGB1), cyclin D1, E1, A2, and phosphorylated NF‐κB (p‐p65) were upregulated in resistant cells. These proteins regulate cell proliferation, migration, and invasion of resistant cells. Depletion of MGB1 decreased cyclin and p‐p65 expression. Cyclin D1 and A2, but not E1 expression, were affected by p‐p65 downregulation. In summary, our results indicate that MGB1 expression is increased in breast cancer cells that have gained resistance to trastuzumab, and suggest that MGB1 promotes aggressiveness through cyclin and NF‐κB regulation.

## Graphical abstract

Trastuzumab is a first‐line therapy for HER2‐positive breast cancer patients, but its efficacy is limited by resistance. Our study revealed that resistant cells showed higher proliferation, invasion, and migration abilities than wild‐type cells. Mammaglobin 1 (MGB1) expression is increased in breast cancer cells that have gained resistance to trastuzumab, and MGB1 promotes aggressiveness through cyclin and NF‐κB regulation.

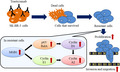

**Abbreviations**
CDKs: cyclin‐dependent kinases
CI: confidence interval
CRC: colorectal cancer cell lines
EGFR: epidermal growth factor receptor
ER: estrogen receptor
FDA: Food and Drug Administration
HCC: hepatocellular carcinoma
HER2: human epidermal growth factor receptor 2
MGB1: mammaglobin 1
MKI67: marker of proliferation Ki‐67
mRNA: messenger ribonucleic acid
MTT assay: 3‐(4,5‐dimethylthiazol‐2‐yl)‐2,5‐diphenyltetrazolium bromide assay
NF‐κB: nuclear factor kappa B
PBS: phosphate buffer saline
PR: progesterone receptor
PTEN: phosphatase and tensin homolog
PVDF: polyvinylidene fluoride
qPCR: quantitative polymerase chain reaction
RNA: ribonucleic acid
SD: standard deviation
SDS: sodium dodecyl sulfate
siRNA: small interfering RNA
TBS‐T: tris‐buffered saline‐Tween

Human epidermal growth factor receptor 2 (HER2), which belongs to the epidermal growth factor receptor (EGFR) superfamily, is a protein involved in one of the most studied signal transduction pathways in cancer [1]. The amplification or overexpression of HER2 is detected in 15–20% of breast [1], 17.9% of gastric and gastroesophageal cancer [2], in 20–30% of some ovarian cancer [3], and is correlated with poor patient survival. The estrogen receptor (ER) and HER2 (*c‐erbB2, HER2/neu*) signaling pathways are the dominant drivers of cell proliferation and survival in the majority (85%) of breast cancer cases [4]. Under normal physiological conditions, HER2 activation is spatially and temporally controlled when the ligand binds to one of the other EGFR family members, leading to heterodimer formation with HER2, which then activates its kinase activity. However, in the abnormal condition, when HER2 is overexpressed, this molecule associates with itself and other EGFR family members and is activated in a ligand‐independent manner [1, 5, 6].

Trastuzumab (Herceptin^®^), a recombinant humanized monoclonal antibody against HER2, has been considered as the first‐gate therapy for HER2‐positive breast cancer patients [7]. However, in some cases, the effectiveness remains low due to acquired or *de novo* resistance. Other anti‐HER2 therapies that have different actions, such as pertuzumab (inhibitor for heterodimerization of HER2 with HER3), lapatinib (intracellular reversible inhibitor of EGFR and HER tyrosine kinase), and ado‐trastuzumab emtansine T‐DM1 (an antibody drug‐conjugated, anti‐HER2 function of trastuzumab and DM1‐induced cytotoxicity), are recommended by the U.S. Food and Drug Administration (FDA) [7, 8]. To improve the therapy, in 2012, the FDA approved the combination of trastuzumab and pertuzumab as a first‐line therapy for HER2+ metastatic breast cancer. Most recently, the trastuzumab‐linked antibody‐drug conjugate, DS‐8201a, has been approved for the treatment of patients with metastatic HER2+ breast cancer who had previously received two or more anti‐HER therapies [7]. The general mechanisms of trastuzumab resistance that have been intensively studied include the following: (a) The difficulty associated with trastuzumab binding to HER2 is caused by a structural mutation in HER2, which generates a truncated p95HER2 isoform [9, 10]; (b) the upregulation of HER2 downstream signaling pathways; (c) signaling through alternate pathways; and (d) failure to stimulate immune‐mediated mechanisms to eradicate tumor cells [10]. The resistance phenomena remain a major obstacle in cancer treatment owing to the complexity and heterogeneity of the mechanism. Therefore, specific markers of cancer resistance need to be explored to enhance the effectiveness of therapy.

Mammaglobin 1 (MGB1), also known as mammaglobin A or *SCGB2A2*, is a member of the secretoglobin family located on a genomic region frequently amplified in breast cancer chromosomes 11q12.3–137,8. Mammaglobin 1 is a promising marker for breast cancer as its specificity has been repeatedly highlighted [11, 12]. Although MGB1 is highly expressed in breast cancer [13, 14], it has also been detected in gynecological malignancies [15]. MGB1 has become a standard marker for detecting disseminated tumor cells in lymph nodes, peripheral blood [16], and micrometastases in bone marrow [17]. The role of MGB1 in cancer progression has been reported in triple‐negative (HER2 negative/ER negative/PR negative) breast cancer cells [18]. However, the role of MGB1 in cancer progression, especially in HER2 positive/ER negative breast cancer‐resistant cells, is still not fully understood.

The cyclin family consists of at least four major types (D, E, A, and B) of the 11 types that have been discovered in mammalian cells [19]. Cyclins are responsible for regulating cell cycle progression by interacting with cyclin‐dependent kinases (CDKs), which govern the stage order from the resting stage (G0 phase) to cell division (M phase) [20]. They also play important roles in cancer progression and metastasis through alternate pathway [20]. Additionally, it has been reported that cyclin is connected to NF‐κB. The most explored connection between NF‐κB activation and cell cycle progression involves cyclin D1. There is also some evidence that NF‐κB may activate the cyclin A promoter [21]. The NF‐κB family consists of five proteins, one of which is RelA/p65. RelA/p65 can generate a heterodimer with NF‐κB1 (p50) [21]. The p65/p50 complex is activated through phosphorylation and translocated to the nucleus to be a critical transcription factor for several genes involved in cancer progression, such as enabling proliferation [22]. Interestingly, NF‐κB is involved in trastuzumab resistance in HER2 positive/ER positive breast cancer cells [23].

Here, we focused on HER2‐positive/ER‐negative breast cancer cells that were chronically treated with trastuzumab to establish resistant cells. We demonstrated that the upregulation of MGB1, which occurred after cells gained resistance, was a crucial factor in cell viability, migration, and invasion abilities by regulating cyclins and NF‐κB in resistant cells.

## Results

### Resistant cells have higher proliferation, invasion, and migration abilities than wild‐type cells

**Fig. 1:**
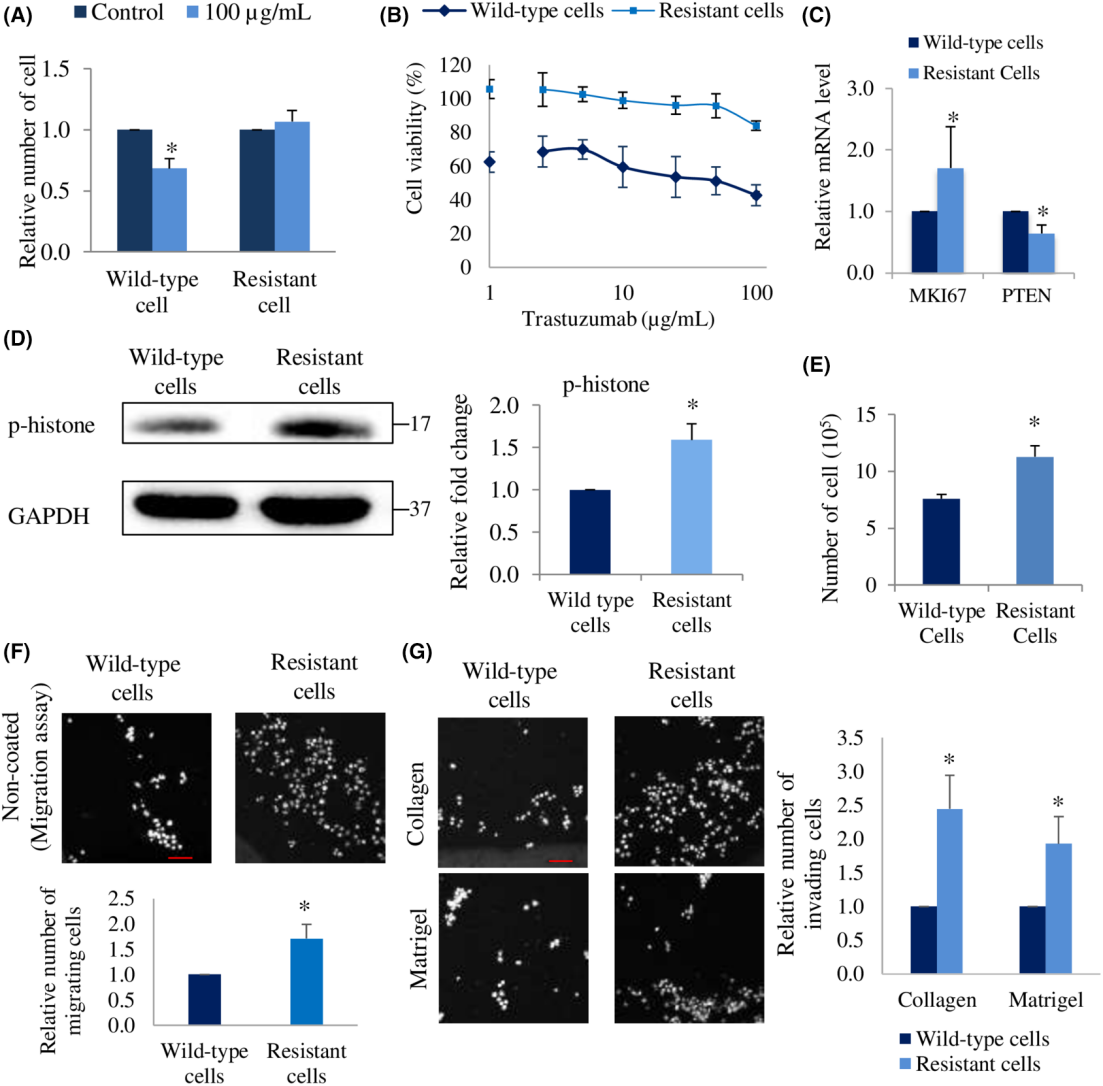
Resistant cells have high proliferation, invasion, and migration abilities. (A) Relative number of SK‐BR‐3 wild‐type and resistant cells after treatment with 100 μg·mL^−1^ of trastuzumab for 5 days. The number of cells relative to each control group (0 μg·mL^−1^ trastuzumab) is shown. (B) Viability of wild‐type and resistant cells after treatment with a series of concentration (0, 1, 2.5, 5, 10, 25, 50, and 100 μg·mL^−1^) of trastuzumab for 5 days. The viability of cells treated with 0 μg·mL^−1^ trastuzumab was used as a basis for the calculation. (C) mRNA level of *MKI67* and *PTEN* in resistant cells relatives to wild‐type cells. β‐actin was used as the control gene. (D) Representative western blots (left), and quantification (right) of p‐histone and GAPDH (loading control) expression in resistant cells relative to wild‐type cells. *N* = 3 experiments, once per experiment. (E) Number of wild‐type and resistant cells after culturing for 5 days in complete culture medium without any treatment. The bar represents standard error mean (SEM), *N* = 3 independent experiments, thrice for each experiment, *P < 0.05, unpaired Welch's *t*‐test. (F) Relative number of migrating and (G) invading cells in resistant cells relatives to wild‐type cells. Bar represents standard error mean (SEM), *N* = 3 independent experiments, thrice for each experiment in A, C, F, G. *Statistical significance in figure A, C, D, F, and G were determined with 95% confidence interval. Scale bar of 100 μm.

First, we established the resistant cells via treatment with 15 μg·mL^−1^ trastuzumab for at least 3 months [24]. To confirm resistance, we investigated the viability of cells that survived after long‐term exposure to trastuzumab (resistant cells), compared to that of untreated cells (wild‐type cells), and found that the viability of resistant cells did not change after exposure to 100 μg·mL^−1^ trastuzumab, whereas that of wild‐type cells decreased by more than 30% (Fig. 1A). Based on the MTT assay, treatment with a high concentration of trastuzumab had markedly greater effects on the viability of wild‐type cells than on resistant cells (Fig. 1B). *MKI67* and p‐histone have been reported as proliferation markers, and a decrease in *PTEN* is used as a resistance marker [25, 26]. Resistant cells showed significantly higher *MKI67* (Fig. 1C) and p‐histone expression (Fig. 1D) and lower *PTEN* mRNA levels than those in wild‐type cells (Fig. 1C). These results demonstrate that resistant cells gained resistance to trastuzumab.

Drug‐resistant cancer cells have been reported to acquire high aggressiveness, which causes poor prognosis in cancer patients [27]. Based on the *MKI67* mRNA level results, we examined the proliferation ability of resistant cells by manual counting. Five days after seeding the cells, the number of resistant cells was higher than that of wild‐type cells (Fig. 1E). Furthermore, they expressed upregulated p‐histone suggesting the high proliferation potential. Additionally, migration and invasion abilities are correlated with cancer progression [28]. To investigate these, we performed invasion and migration assays on collagen‐ or Matrigel‐coated membranes and on noncoated membrane trans‐wells, respectively. After incubating the cells for 18–20 h, we observed that the invasion and migration abilities of resistant cells significantly increased compared to those of wild‐type cells (Fig. 1F,G). These results suggest that after breast cancer cells gain trastuzumab resistance, their proliferation, invasion, and migration abilities are enhanced.

### MGB1 is upregulated in resistant cells and is critical for the aggressiveness of these cells

**Fig. 2:**
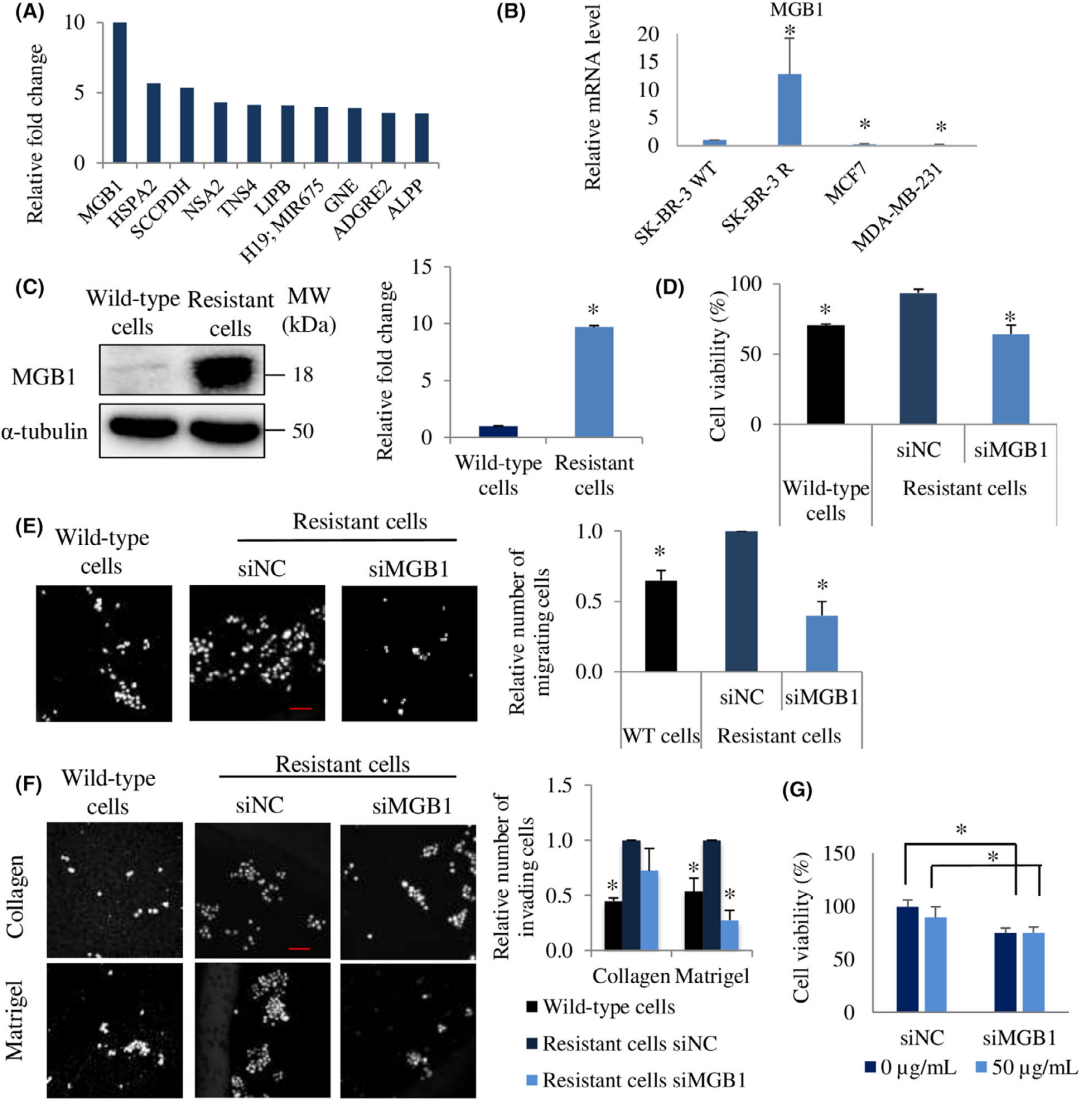
*MGB1* is upregulated in resistant cells and is critical for their aggressiveness. (A) The top 10 upregulated genes (mRNA level) of resistant cells relative to wild‐type cells in the microarray assay. *N*  = 1, experiment. (B) Comparison of the *MGB1* mRNA level in SK‐BR‐3 (wild‐type cells, HER2 positive/ER negative), SK‐BR‐3 R (resistant cells), MCF7 (HER2 negative/ER positive), and MDA‐MB‐231 (HER2 negative/ER negative) cells. The bar represents mean with SEM, *N*  = 3 independent experiments, thrice for each experiment, *statistical significance with CI of 95%. (C) Representative western blots (left) and quantification (right) of MGB1 and α‐tubulin (loading control) in resistant cells relative to wild‐type cells. The bar represents mean with SEM, *N*  = 3, once per experiment, *statistical significance with CI of 95%. (D) Cell viability, the bar represents mean with SEM, *N*  = 3 independent experiments, thrice for each experiment, *P  < 0.05, unpaired Welch's *t*‐test. (E) Migration, and (F) invasion of resistant cells after transfection with siMGB 1 relative to the negative control (siNC). The bar represents mean with SEM, *N*  = 3 independent experiments, thrice for each experiment, *statistical significance with CI of 95%. Scale bar of 100 μm. (G) Viability of resistant cells after transfection with siMGB1 or siNC followed by trastuzumab treatment (50 μg·mL ^−1^ for 4 days). The bar represents mean with SEM, *N* = 2 independent experiments, thrice per experiment with *P value < 0.05 unpaired Student's *t*‐test.

Next, to investigate key molecules involved in the proliferation, migration, and invasion abilities of resistant cells, we performed an RNA microarray. The results demonstrated that *MGB1* expression was upregulated up to 10‐fold in resistant cells compared to that in wild‐type cells (Fig. 2A). To evaluate the cell line specificity for *MGB1*, we detected the mRNA expression of *MGB1* in other breast cancer cells, MCF7 (HER2 negative/ER positive/PR positive) and MDA‐MB‐231(HER2 negative/ER negative/PR negative). We found that SK‐BR‐3 cells had higher *MGB1* expression than that in other cells (Fig. 2B). Moreover, when the cells gained trastuzumab resistance, the mRNA and protein levels of *MGB1* were significantly elevated (Fig. 2B,C). To examine the role of MGB1 in the resistant cells, we knocked down *MGB1* in resistant cells using siRNA. As the proliferation ability of resistant cells was higher than that of wild‐type cells, we investigated the effect of MGB1 on cell proliferation using MTT assay. *MGB1* knockdown was found to significantly decrease the viability of resistant cells such that their cell viability was similar to that of wild‐type cells (Fig. 2D). The transwell assay was performed to compare the migration and invasion abilities of control and MGB1‐depleted cells. The migration assay and Matrigel‐coated invasion assay revealed that silencing MGB1 expression decreased the migration and invasion abilities of resistant cells, respectively (Fig. 2E,F). However, a significant decrease was not observed in the collagen‐coated membrane invasion assay (Fig. 2F). These results suggest that MGB1 is a key molecule for the aggressiveness of resistant cells.

**Fig. 3:**
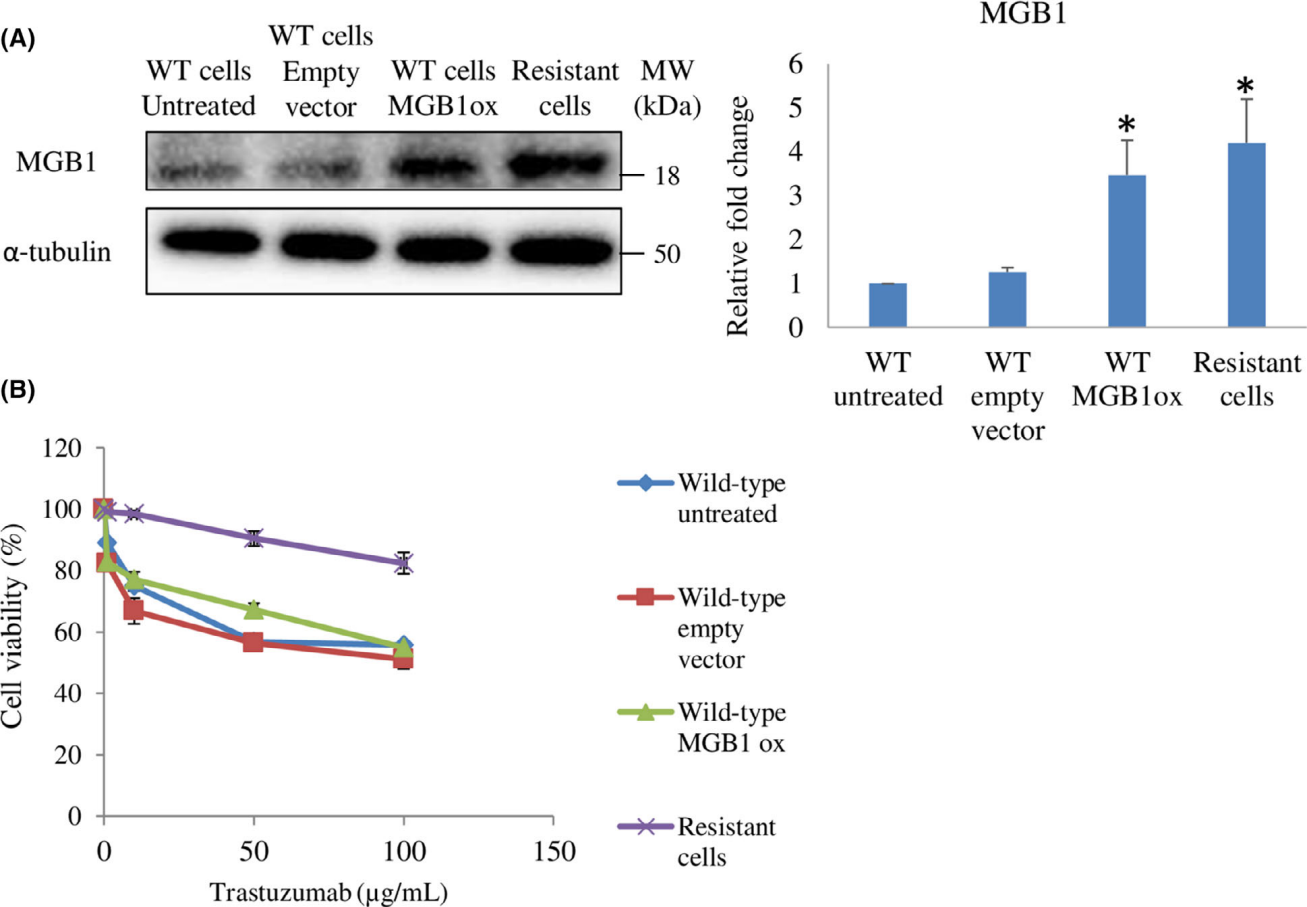
*MGB1* overexpression in wild‐type cells does not promote trastuzumab resistance. (A) Representative western blots (left), and quantification (right) of *MGB1* and α‐tubulin (loading control) expression in wild‐type cells (untreated, empty‐vector, and *MGB1*‐overexpressing (MGB1ox) and resistant cells relative to wild‐type cells untreated. *N* = 3 experiments, once for each experiment. (B) Viability of wild‐type cells (untreated, empty‐vector, and MGB1ox) and resistant cells after the treatment with different concentrations (1, 10, 50, and 100 μg·mL^−1^) of trastuzumab for 5 days. The viability of treated cells was calculated relative to that of untreated cells. The bar represents the mean with SEM, *N* = 3 independent experiments, thrice for each experiment, *statistical significance was determined with 95% confidence interval.

Subsequently, to clarify whether MGB1 regulates the survival of resistant cells after trastuzumab treatment, *MGB1*‐depleted resistant cells were exposed to 50 μg·mL^−1^ trastuzumab for 4 days. There was no difference in the *MGB1*‐knocked‐down resistant cells' viability with and without trastuzumab (Fig. 2G). Additionally, to investigate the role of *MGB1* in the resistance process, we induced *MGB1* overexpression in wild‐type cells, which expressed *MGB1* at lower levels than that in resistant cells. *MGB1* wild‐type cells overexpressing *MGB1* approximately threefold (MGB1ox wild‐type cells) were successfully generated, as shown in Fig. 3A. MGB1ox wild‐type cells were exposed to trastuzumab at concentrations up to 100 μg·mL^−1^, and we found that *MGB1* overexpression did not induce trastuzumab resistance (Fig. 3B). These findings may suggest that an increase in *MGB1* expression may occur in breast cancer cells after developing trastuzumab resistance in a trastuzumab‐independent manner.

### MGB1 regulates cyclins and p‐p65 expression in resistant cells

**Fig. 4:**
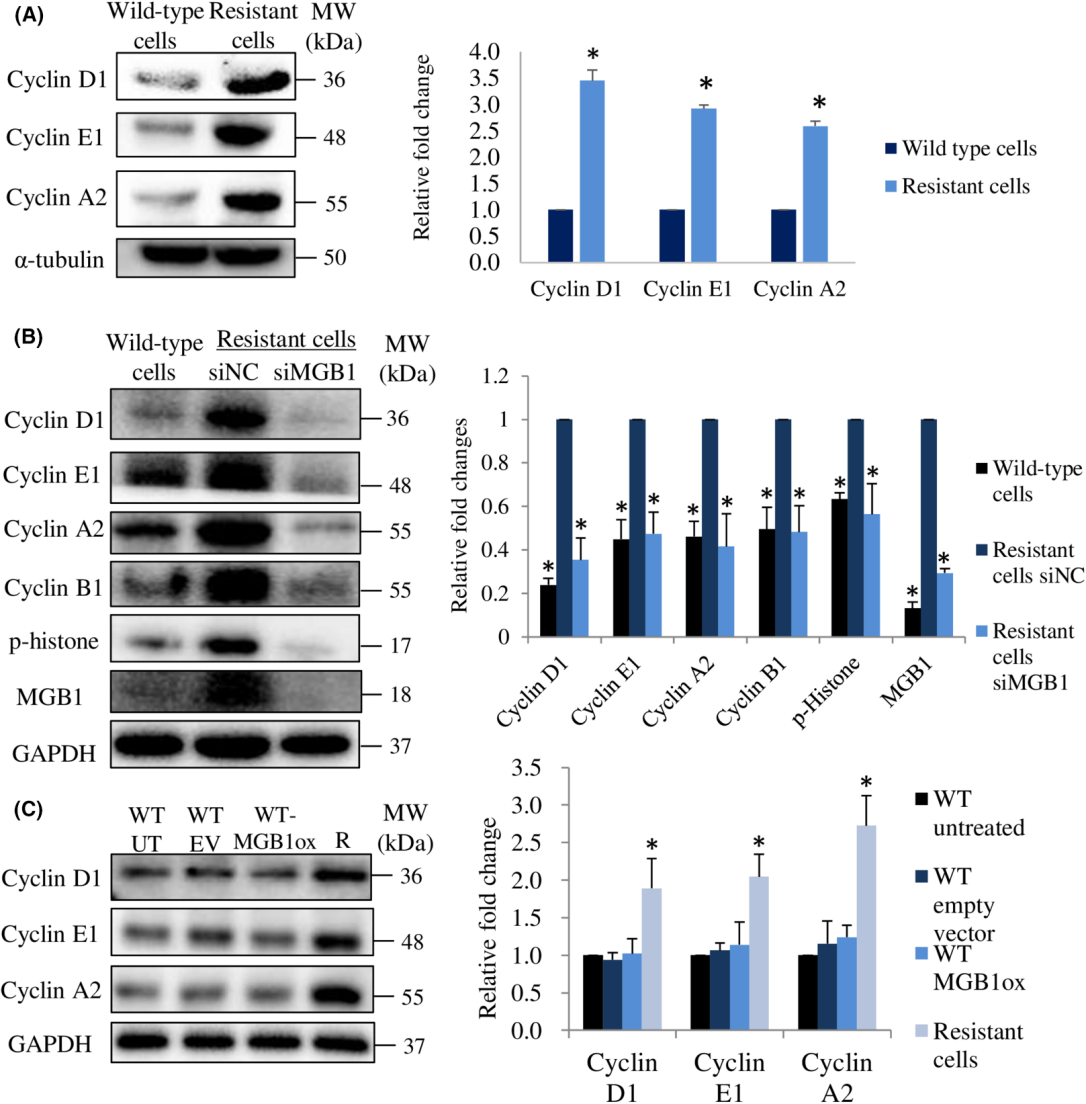
MGB1 regulates cyclin expression in resistant cells but not in wild‐type cells overexpressing MGB1. (A) Representative western blots (left) and quantification (right) of cyclin D1, cyclin E1, cyclin A2, and α‐tubulin (loading control) in resistant cells relative to wild‐type cells. (B) Representative western blots (left) and quantification (right) of cyclin D1, cyclin E1, cyclin A2, cyclin B1, p‐histone, MGB1, and α‐tubulin (loading control) in resistant cells after siMGB1 transfection relative to negative control (siNC). (C) Representative images of western blots (left) and quantification (right) of cyclins and GAPDH (loading control) in untreated wild‐type cells (WT UT), wild‐type cells with empty vector (WT‐EV), wild‐type cells with *MGB1* overexpression (WT‐MGB1ox), and resistant cells (R). All statistical analysis in this figure is shown as the bar represents the mean with SEM, *N* = 3 independent experiments, once for each experiment. *Statistical significance was determined with 95% confidence interval.

Our results showed that *MKI67* and p‐histone expression were upregulated in resistant cells. Upregulation of these molecules was strongly related to the elevation of cell proliferation [29]. Therefore, to investigate the detailed mechanism in resistant cells, we detected marker proteins of each phase in the cell cycle process. We found that resistant cells showed higher expression of cyclins (cyclin D1, cyclin E1, and cyclin A2) than wild‐type cells (Fig. 4A). Then, we investigated the role of MGB1 in cyclin expression in resistant cells. We found that the expression of cyclins and p‐histone was significantly downregulated by MGB1 depletion (Fig. 4B). However, an increase in cyclin expression was not observed in MGB1ox wild‐type cells (Fig. 4C).

**Fig. 5:**
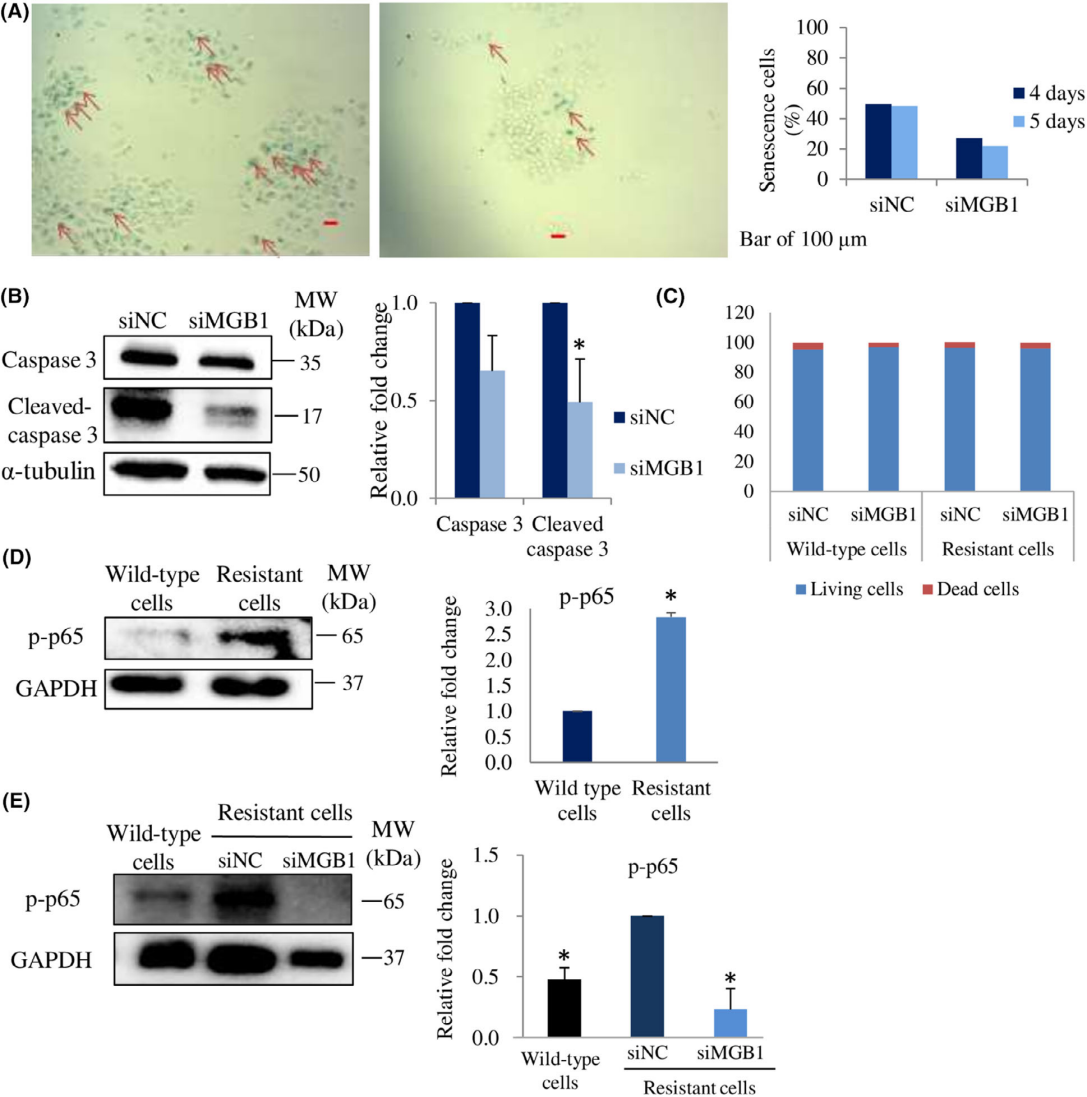
MGB1 does not regulate apoptosis and senescence cells but p‐p65 expression. (A) Representative images (left) of senescence cells (red arrow), and their quantification (number of senescence cells per 100 cells) 4 and 5 days posttransfection (right). Scale bar of 100 μm. (B) Representative western blots (left), and quantification (right) of caspase 3, cleaved caspase 3, and α‐tubulin (loading control) expression in resistant cells after siMGB1 transfection, relative to that of the negative control (siNC). (C) Graph bars showing the percentage of living cells and dead cells in wild‐type and the resistant group treated with siMGB1. (D) Representative western blots (left) and quantification (right) of p‐p65 and GAPDH (loading control) expression in resistant cells relative to that of wild‐type cells. (E) p‐p65 and GAPDH (loading control) expression in resistant cells after siMGB1 transfection, relative to that of the negative control (siNC). All statistical analysis in this figure is shown as the bar represents the mean with SEM, *N* = 3 independent experiments, once for each experiment. *Statistical significance was determined with 95% confidence interval.

To determine other mechanisms that reduce cell viability caused by *MGB1* knock‐down in resistant cells, we evaluated cleaved‐caspase 3 expression for the detection of apoptosis and β‐galactosidase activity to analyze senescence. SK‐BR‐3 cells naturally undergo senescence if they are not passaged within 4 days. Therefore, in the senescence assay, we deliberately prolonged the cell culture period to ensure that senescence did not occur in *MGB1*‐depleted resistant cells. We found that cleaved‐caspase 3 expression and β‐galactosidase activity decreased in *MGB1*‐knocked‐down resistant cells (Fig. 5A,B). Cell death assessment using trypan blue assay supports the western blotting results, as the number of dead cells did not increase after *MGB1*‐knockdown (Fig. 5C). We suggest that the decrease in cell viability by *MGB1* knockdown in resistant cells was due to the prevention of cell proliferation rather than apoptosis or senescence induction. It has been reported that trastuzumab does not induce senescence in SK‐BR‐3 wild‐type cells [30]. This phenomenon occurs even if SK‐BR‐3 cells confer trastuzumab resistance.

One of the crucial factors reported to be involved in breast cancer cell malignancies is NF‐κB [31, 32]. Based on this finding, we investigated the expression and role of the most‐studied NF‐κB family member, p65 [21, 22] especially phosphorylated‐p65 (p‐p65), the active state of p65 protein, in resistant cells. The expression of p‐p65 in resistant cells was significantly higher than that in wild‐type cells (Fig. 5D). Then, we examined whether MGB1 expression was involved in p‐p65 expression. The results showed that a decrease in MGB1 downregulated p‐p65 expression (Fig. 5E). These findings suggest that MGB1 contributes to the regulation of cyclins and p‐p65 expression in resistant cells.

### Cyclins regulate MGB1‐dependent aggressiveness in resistant cells

**Fig. 6:**
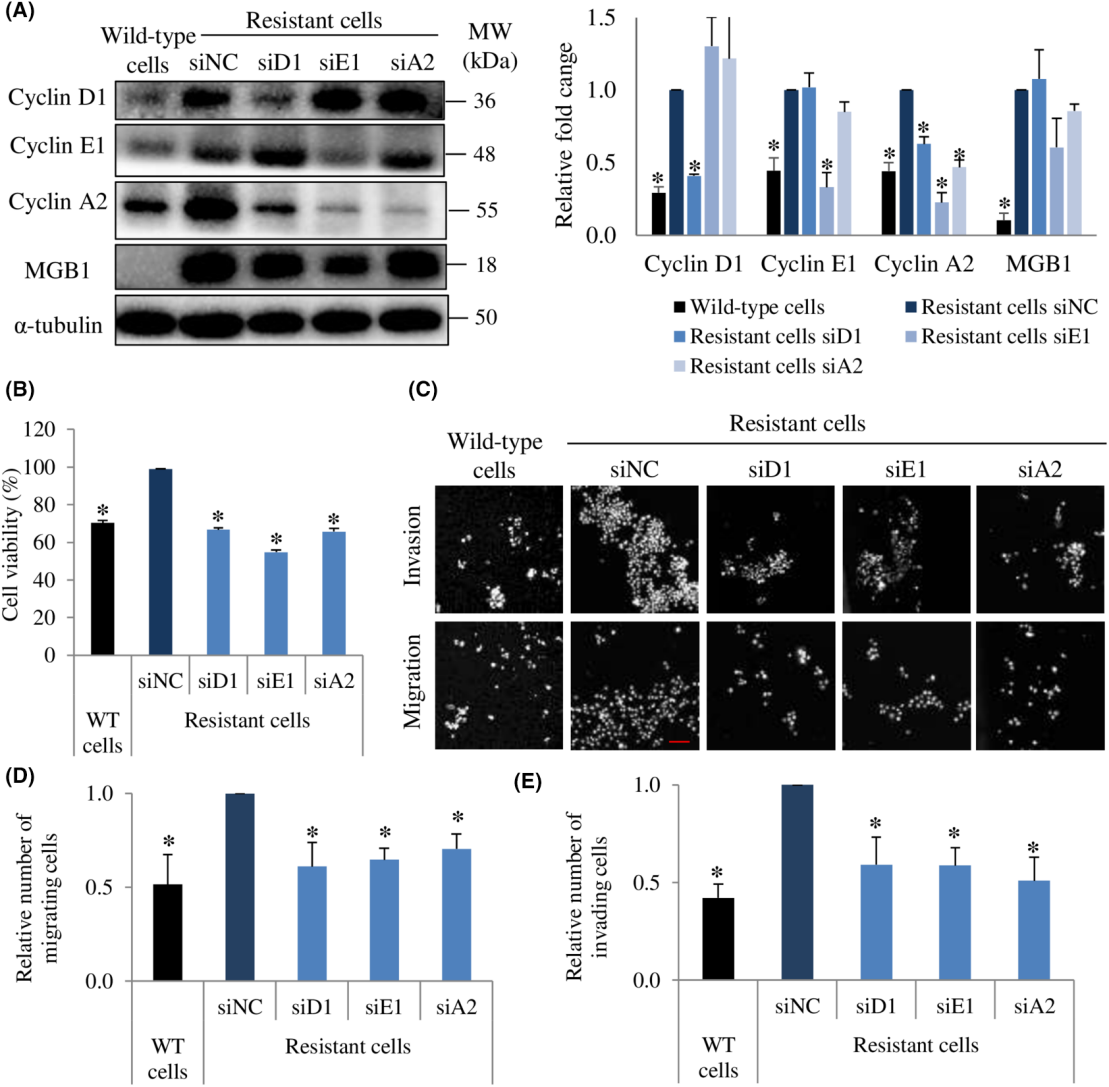
Cyclin D1, E1, and A2 are involved in MGB1‐regulated aggressiveness in resistant cells. (A) Representative western blots (left) and quantification (right) of cyclin D1, cyclin E1, cyclin A2, and α‐tubulin (loading control) in resistant cells after siCyclin D1 (siD1), siCyclin E1 (siE1), and siCyclin A2 (siA2) transfection relative to the negative control (siNC). *N* = 3 independent experiments, once per experiment. (B) Cell viability. Bar represents mean with SEM, *N* = 3 independent experiments, thrice for each experiment, *P value <0.05 unpaired Welch's *t*‐test. (C) Representative figure of migration and invasion assay with 20× magnification. Scale bar of 100 μm. (D) Quantification of migration. *N* = 3 independent experiments, thrice for each experiment, and (E) invasion of resistant cells after transfection with siD1, siE1, and siA2 relative to the negative control (siNC). The bar represents the mean with SEM, *N* = 3 independent experiments, thrice for each experiment. *Statistical significance of figure A, D, and E was determined with 95% confidence interval.

Numerous studies have reported that in addition to cell cycle progression, cyclin D1 [33, 34, 35], cyclin E1 [36], and cyclin A2 [37, 38] are also involved in the regulation of cell migration and invasion. Based on the upregulation of cyclin in resistant cells, we investigated the role of cyclin D1, cyclin E1, and cyclin A2 in the regulation of viability, migration, and invasion abilities. We successfully knocked down their expression by siRNA (Fig. 6A). Cell viability was decreased by silencing each cyclin in the resistant cells (Fig. 6B), as well as migration and invasion abilities (Fig. 6C–E). These findings suggested that the downregulation of cyclin D1, cyclin E1, and cyclin A2 decreased the viability, migration, and invasion ability of resistant cells. Next, we examined whether each cyclin regulated the expression of another cyclin. Cyclin D1 and cyclin E1 expression did not change when other cyclins were downregulated, whereas cyclin A2 expression level was decreased by silencing cyclin D1 or E1 (Fig. 6A). We also found that MGB1 expression was not affected by a decrease in cyclin expression (Fig. 6A). These results indicate that MGB1 is an upstream regulator of cyclins in trastuzumab‐resistant HER2 positive/ER negative breast cancer cells.

### P‐p65 triggers MGB1‐regulated aggressiveness in resistant cells through the induction of cyclins

**Fig. 7:**
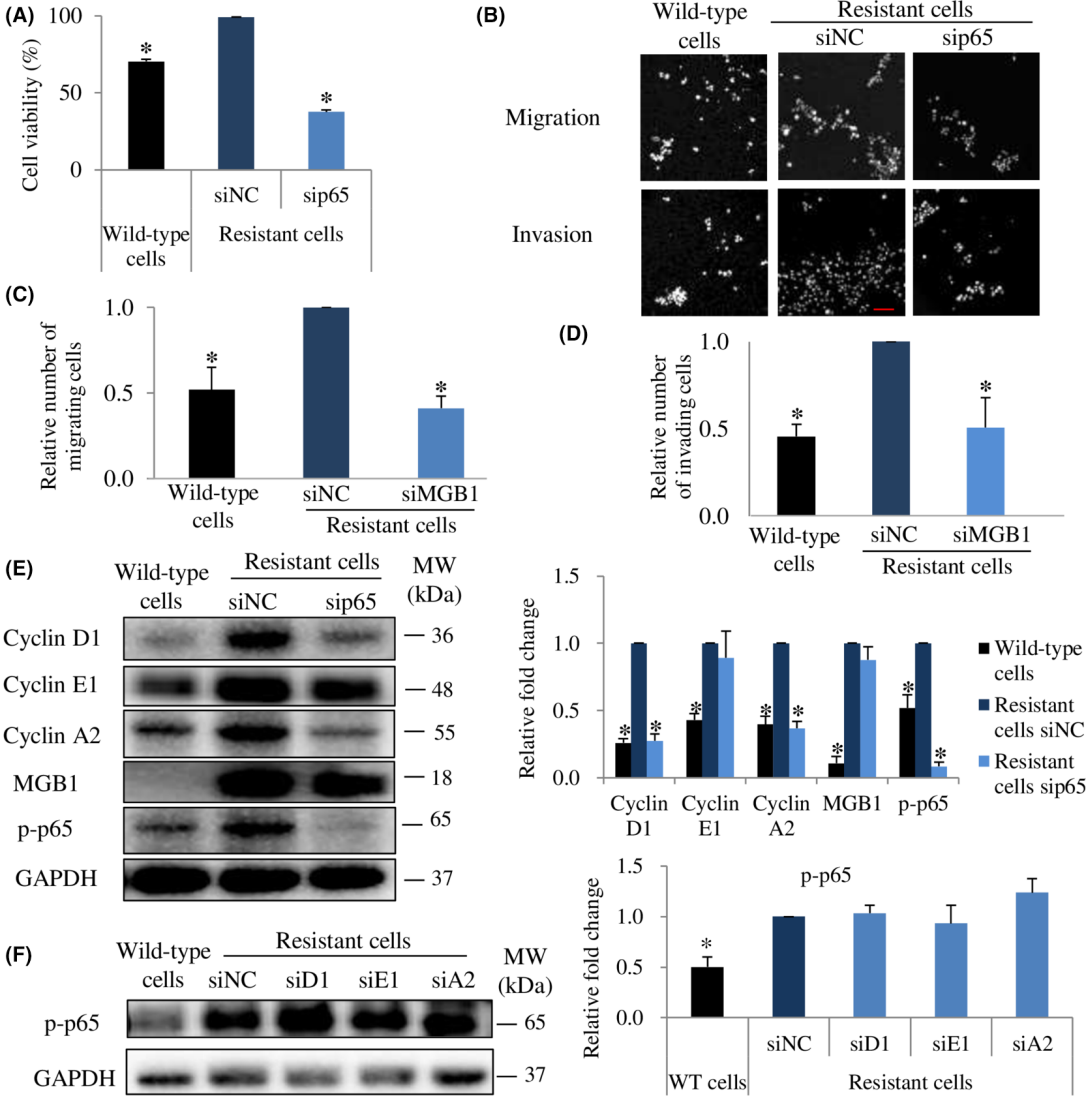
P‐p65 is involved in MGB1‐regulated aggressiveness in resistant cells through cyclin regulation. (A) Cell viability, Bar represents mean with SEM, *N* = 3 independent experiments, thrice for each experiment, *P value <0.05 unpaired Welch's *t*‐test. (B) Representative figure of migration and invasion assay with 20× magnification. Scale bar of 100 μm. (C) Quantification of migration. *N* = 3 independent experiments, thrice for each experiment, and (D) invasion of resistant cells after transfection with sip65 relative to the negative control (siNC). *N* = 3 independent experiments, thrice for each experiment. (E) Representative western blots (left) and quantification (right) of cyclin D1, cyclin E1, cyclin A2, MGB1, p‐p65, and GAPDH (loading control) in wild‐type cells, siNC and siMGB1‐resistant cells, relative to siNC resistant cells. *N* = 3 independent experiments, once for each experiment. (F) Representative western blots (left) and quantification (right) of p‐p65 and GAPDH (loading control) in wild‐type cells, and resistant cells after siD1, siE1, and siA2 transfection relative to the resistant cells negative control. The bar represents the mean with SEM, *N* = 3 independent experiments, once for each experiment. *Statistical significance was determined with 95% confidence interval.

Activation of NF‐κB through HER2 signaling is essential for HER2‐mediated cancer resistance [39]. Since p‐p65 was upregulated in resistant cells, we investigated the viability, migration, and invasion abilities of p65‐depleted resistant cells. We discovered that cell viability decreased by more than 60% (Fig. 7A). Moreover, migration and invasion abilities of resistant cells decreased significantly by silencing the p65 gene (Fig. 7B–D). Additionally, it has been known that NF‐κB regulates cyclin activation [21]. Therefore, we examined the relationship between p‐p65 and cyclin expression. The downregulation of p65 decreased cyclin D1 and cyclin A2 expression but did not decrease cyclin E1 and MGB1 expression (Fig. 7E). Meanwhile, the downregulation of each cyclin had no significant effect on p‐p65 expression (Fig. 7F). These findings suggest that p‐p65 regulates cyclin D1 and A2, whereas cyclins are not critical for p‐p65 expression.

### Antibody‐dependent cellular cytotoxicity may not change due to resistance and MGB1 depletion

Antibody‐dependent cellular cytotoxicity (ADCC) and antibody‐dependent cellular phagocytosis (ADCP) are the dominant immune‐based antitumor effects of trastuzumab. An important process in ADCC and ADCP is the binding of trastuzumab to the HER2 receptor. Therefore, ADCC and ADCP can still occur if tumor cells express HER2 target antibody‐binding epitopes [40].

**Fig. 8:**
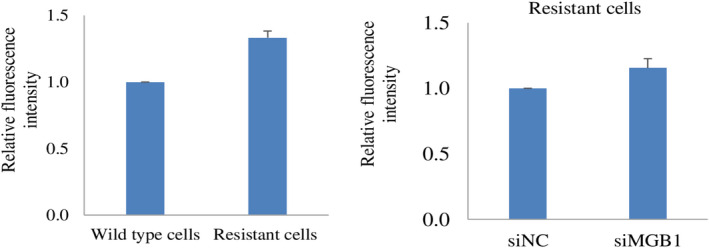
Detection of HER2 receptor in resistant cells and siMGB1‐treated resistant cells. Detection of HER2 receptor ectodomain by trastuzumab‐binding assay in resistant cells relative to wild‐type cells (left), and in siMGB1‐resistant cells relative to the negative control (right). The fluorescence intensity values indicate the binding of trastuzumab to the HER2 receptor. Graph bars represents mean ± SEM, *N* = 3 independent experiments, thrice for each experiment. No significant differences were observed in any of the comparisons.

Here, we performed a trastuzumab‐binding assay to observe the ability of trastuzumab to bind to the HER2 receptor in resistant cells and *MGB1*‐depleted resistant cells. Our results demonstrated that resistance to trastuzumab did not affect its binding to the resistant cells. Likewise, *MGB1* depletion did not affect trastuzumab‐binding efficiency (Fig. 8), indicating that ADCC and ADCP may still occur in resistant cells even when *MGB1* is silenced.

## Discussion

**Fig. 9:**
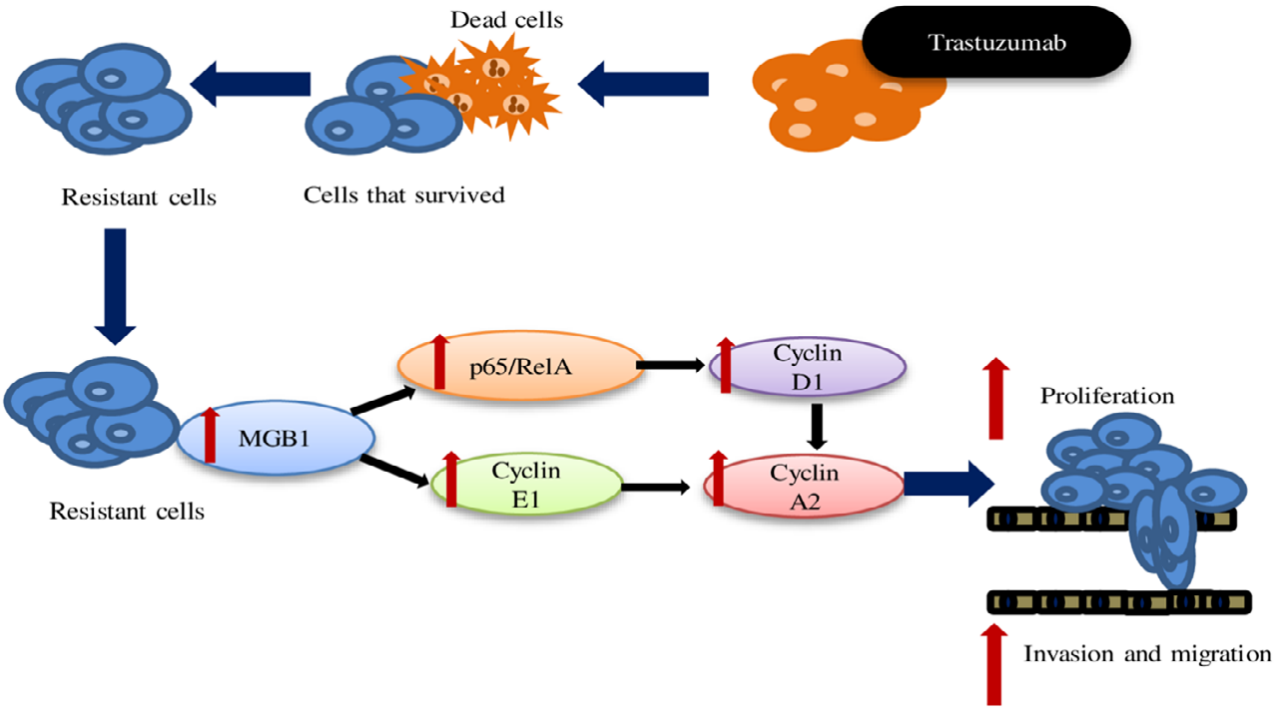
Schematic depicting how MGB1 regulates the progression of resistant cells through cyclin and NF‐κB regulation. The surviving cells after long‐term treatment with trastuzumab showed resistance. MGB1 is upregulated in resistant cells and regulates progression (cell proliferation, migration, and invasion ability; proliferation is critical for cell viability) through the induction of NF‐κB‐dependent cyclin D1 expression and cyclin E1 expression. Cyclin D1 and E1 expression affected cyclin A2 expression.

Briefly, we demonstrated that resistant cells MGB1 regulated proliferation, migration, and invasion abilities through cyclin and p‐65 signaling (Fig. 9). In this study, we established trastuzumab‐resistant cells by chronic treatment with trastuzumab. This approach mimics that used in the clinic, where acquired resistance rises gradually [41] in long‐term administration of trastuzumab. We found that HER2 positive/ER negative breast cancer cells enhanced MGB1 expression up to 10‐fold after trastuzumab resistance. This result was supported by a previous report, which also showed that MGB1 (*SCGB2A2*) is upregulated in trastuzumab‐resistant HER2‐overexpressed breast cancer cells [42]. However, as there is a lack of evidence regarding the relationship between HER2 and MGB1, it should be further explored. Although it has been reported that MGB1 is highly expressed in various types of breast cancer cell including HER2 positive/ER negative type [18], the role of MGB1 in this type has not been reported.

Overcoming cell cycle arrest is one of the mechanisms of trastuzumab resistance [9]. Increment of all major cyclin (D1, E1, and A2) in resistant cells may therefore render cells independent of cell cycle arrest mediated by trastuzumab. A previous study reported that the downregulation of MGB1 decreased the viability of triple‐negative breast cancer cells [18]. This finding was consistent with our results, which used different types of breast cancer cells. Cyclins play an essential role in cell cycle regulation. However, there is no evidence of a relationship between MGB1 and cyclins. Our findings revealed that MGB1 silencing downregulated cyclin expression. The decrease in MGB1 and cyclin D1, E1, and A2 decreased the viability of resistant cells. Additionally, MGB1 and cyclins contributed to the regulation of the migration and invasion abilities of resistant cells. A previous study reported that the upregulation of cyclin A2 enhanced the migration of hepatocellular carcinoma (HCC), while its downregulation decreased the migration of DLD‐1 and SW620 colorectal cancer cell lines (CRC) [38, 43]. Cyclin D1 enhances cell migration and invasion activity in fibroblasts by regulating RhoA [33] as well as tumor cells [34, 35, 44]. These findings are consistent with our results. However, cyclin A2 depletion enhanced the migration of fibroblasts and the invasiveness of CRCs and triple‐negative breast cancer cells by downregulating RhoA activity [37]. These contradictory results are possibly due to the difference in the molecular profile of each cell, or the type of matrix used for the experiments. Three studies used Matrigel, while the latest study used collagen as the matrix. In some cases, the collagen and Matrigel matrices have opposite roles [45, 46]. Contradicting results were also found for cyclin D1 function, even though the same breast cancer cell line was used [35, 47]. The expression levels of cyclin A and E were correlated with each other, but not with cyclin D1 [48]. Cyclins have diverse functions, however, their regulatory function involves various signaling pathways and different conditions [21, 37, 38, 49]. NF‐κB regulates cyclin D1 and cyclin E1 expression to regulate glioma cell growth and invasion [49]. A previous study reported that a lack of MGB1 decreased NF‐κB transactivation by increasing IκB‐α (inhibitor of NF‐κB alpha) in triple‐negative breast cancer cells [18]. These previous results support our finding that the regulation of NF‐κB and cyclin through MGB1 plays a crucial role in the aggressiveness of resistant cells.

Based on a mouse model, cyclin redundancy has been reported to occur in cell cycle regulation [19]. In the absence of D‐type cyclin, the cell cycle process can still be run by the presence of cyclin E or cyclin A. Cyclin D and cyclin A can substitute for the absence of cyclin E to maintain cell survival [19]. In contrast, cyclin A2 is one of the most nonredundant cyclins [19]. These findings support our data, which showed that a decrease in cyclin D1 through NF‐κB, or cyclin E1 decreased cyclin A2 expression. Through this regulation, at least two types of cyclins (D1 and A2 or E1 and A2) decreased the viability, migration, and invasion abilities of resistant cells. Meanwhile, the depletion of cyclin A2 did not influence other cyclins, NF‐κB, and MGB1 expression, however, single cyclin A2 depletion decreased cell viability, migration, and invasion abilities. Further research should be conducted to determine whether the depletion of a single cyclin can have a similar function. Moreover, the correlation of each cyclin in resistant cells should be further explored.

In summary, we demonstrated that the upregulation of MGB1 was induced by trastuzumab resistance in HER2‐positive/ER negative breast cancer cells, and MGB1 increased cell viability, migration, and invasion abilities by upregulating cyclin and NF‐κB expression. We observed that *MGB1* overexpression in wild‐type cells did not trigger cyclin upregulation, indicating no correlation between cyclin and *MGB1* expression. This may indicate that regulation of cyclins expression by *MGB1* only occurs after the cells acquire resistance to trastuzumab.

One of the recent strategies of trastuzumab combination therapy is immune‐based biomarkers [7]. Further, MGB1 has previously been developed as a vaccine for breast cancer [50]. Therefore, our findings indicate that MGB1 could be a prospective marker for detecting resistance in breast cancer patients receiving long‐term trastuzumab treatment. Failure to stimulate immune‐mediated mechanisms to eliminate tumor cells is one of the characteristics of trastuzumab‐resistant cancer cells [10]. Similar to our results (Fig. 8), it has been reported that even after relapse upon treatment with trastuzumab, resistant cells still overexpress HER2 [51]. Additionally, our results revealed that ADCC and ADCP might occur even if *MGB1* is depleted. However, further experiments should be conducted to confirm this hypothesis. In any case, we can conclude that *MGB1* could be a promising therapeutic target for HER2‐positive breast cancer patients with trastuzumab resistance.

## Materials and methods

### Cell culture

SK‐BR‐3‐luc, HER2‐overexpressing breast cancer cells without hormone receptors (JCRB Cell Bank, 1627.1, Japan), MCF7 (ATCC, Manassas, VA, USA), and MDA‐MB‐231 (ATCC) cells were cultured in low‐glucose Dulbecco's modified Eagle's medium (Sigma‐Aldrich Co. LLC, St Louis, MO, USA) supplemented with 10% fetal bovine serum (Sigma‐Aldrich Co. LLC) and 1% antibiotic/antimycotic solution (Sigma‐Aldrich Co. LLC). The cells were cultured in a humidified incubator at 37 °C and 5% CO_2_. Trastuzumab‐resistant SK‐BR‐3‐luc cells were obtained as previously described [24]. A mycoplasma test (Venor™GeM Mycoplasma Detection Kit, PCR‐based, Sigma‐Aldrich Co. LLC) was conducted every 6 months; the cells were confirmed to be negative for mycoplasma contamination.

### Transfection with small interfering RNA (siRNA)

siRNA duplexes and a negative control (nonspecific RNA) were synthesized using the *in vitro* transcription T7 kit (Takara, Otsu, Japan). Cells were transfected with specific siRNA duplexes using Lipofectamine RNAiMAX reagent (Invitrogen, Carlsbad, CA, USA). The target sequences are listed in Table 1. Cells (3 × 10^5^) were seeded in a 6 well‐culture plate. After 48 h of incubation, the medium was changed to antibiotic‐free medium and specific siRNA (1 pmol/100 μL medium/each well in 96‐well plate for MTT assay, 5 or 10 pmol/2 mL medium/each well in 6‐well plate for another assay). After 48 h of transfection, siRNA was discarded, and the cells were incubated in complete medium for 24 h.

**Table 1:** List of siRNA target sequences.

Target name	Target sequence
MGB1	CACTACAAATGCCATAGATGAAT
Cyclin D1	TCCTACGATACGCTACTATAAAG
Cyclin E1	CAAGGAAAAGACATACTTAAG
Cyclin A2	ATCTAAGCATATCTGAATACAGT
p65	CTCTTTCTACTCTGAACTAATAA
NC	AATAGCCCATATGGAAACTGA (anti‐sense sequence)

### Quantitative polymerase chain reaction (qPCR)

RNA extraction was conducted according to the manufacturer's protocol (FG‐80250, FastGene™ RNA Basic Kit/Basic Kit, Nippon Genetics, Tokyo, Japan), and reverse transcription reaction was performed using ReverTra Ace qPCR RT Master Mix (Toyobo, Osaka, Japan). qPCR was conducted using SYBR Green (KAPA SYBR Fast qPCR kit, Nippon Genetics) and an Applied Biosystems StepOnePlus qPCR machine (Thermo Fisher Scientific, Tokyo, Japan). β‐actin was used as the control. Primer sequences for qPCR are listed in Table 2.

**Table 2:** List of primer sequences.

Target name	Forward	Reverse
MGB1	CAAGACAATCAATCCACAAGTGTCTAAGAC	CAGAGTTTCATCCGTTTGGTTAAGAAAACATTC
MKI67	ATCGTCCCAGTGGAAGAGTTG	TCGACCCCGCTCCTTTTGATAG
PTEN	GCGGAACTTGCAATCCTCAG	ACTTGTCTTCCCGTCGTGTG
Cyclin D1	GCTGCGAAGTGGAAACCATC	TTCTGTTCCTCGCAGACCTC
Cyclin E1	GCAGGATCCAGATGAAGAAATG	CTCTCTATTTGCCCAGCTCAG
Cyclin A2	TGGATGGTAGTTTTGAGTCACC	AACCAGTCCACGAGGATAGC
p65	CGCTGCATCCACAGTTTCCAGA	TAGTCCCCACGCTGCTCTTCTA
β‐Actin	TGGGACGACATGGAGAAAATCTG	AGGTCTCAAACATGATCTGGGTC

### MTT assay

Cell viability was measured using the 3‐(4,5‐dimethylthiazol‐2‐yl)‐2,5‐diphenyltetrazolium bromide (MTT) assay. Briefly, cells (3 × 10^3^ cells per well) were cultured in a 96‐well plate. After 48 h of incubation, the medium was replaced with the appropriate treatment. After incubation, the medium was replaced with MTT (0.5 mg·mL^−1^) (Sigma, Darmstadt, Germany) and incubated for approximately 4 h at 37 °C and 5% CO_2_. The reaction was stopped with 10% sodium dodecyl sulfate (SDS) in 0.01 N HCl solution and incubated overnight under dark conditions to dissolve the formazan salt. The cell absorbance was measured using a microplate reader (Bio‐Rad Laboratories, Inc., Tokyo, Japan) at 595 nm. The cell absorbance value was converted to the percentage of viable cells.

### Trans‐well invasion and migration assay

The invasion assay was performed by seeding 5 × 10^4^ cells on the top of an 8.0 μm pore insert (24 well insert, Corning Inc., Corning, NY, USA), which was coated with type 1 collagen (Cell matrix 1‐P Nitta Gelatine, Osaka, Japan), diluted 10× with pH 3 hydrochloric acid or Growth Factor Reduced Matrigel (cat. 354230, Corning, NY, USA) diluted 1 : 6 with cold phosphate buffer saline (PBS). Noncoated inserts were used to observe migration ability. In the upper chamber, serum‐free medium was added, and medium supplemented with 10% FBS was added to the lower wells. The cells were then incubated for 18–20 h. Cells were fixed with 4% paraformaldehyde for 5 min and washed with PBS. Cells on the upper surface of the membrane were removed using a cotton swab. Cells on the lower surface were stained with Hoechst (nuclear dye). Invading and migrating cells were observed using a confocal laser scanning microscope (A1R Confocal Imaging System, Nikon Intech Co., Tokyo, Japan) with a 20× objective lens. The number of cells was counted using the imagej software.

### Western blotting

The samples were prepared as previously described [52] without ultrasonic fragmentation. The lysates were run on 8%, 10%, 12%, or 13% SDS‐polyacrylamide gels and then transferred to polyvinylidene fluoride (PVDF) membranes. The membrane was incubated with 5% or 10% skim milk in Tris‐buffered saline‐Tween (TBS‐T) for 1 h. The membranes were then incubated with the following specific diluted primary antibodies: anti‐GAPDH (1 : 100 000, AM4300, Thermo Fisher Scientific Baltics UAB, Vilnius, Lithuania), anti‐α‐tubulin (1 : 50 000；T5168, Sigma‐Aldrich, Tokyo, Japan), anti‐cyclin A2 (1 : 2000; cat. #4656; Cell Signaling Technology, Danvers, MA, USA), anti‐cyclin B1 (1 : 3000; cat. #4138; Cell Signaling Technology), anti‐cyclin D1 (1 : 1000; cat. #2978; Cell Signaling Technology), anti‐cyclin E1 (1 : 10 000; cat. #4129; Cell Signaling Technology), anti‐p‐histone (1 : 2000; cat. #3377; Cell Signaling Technology), anti‐mammaglobin A (1 : 1000; ab150359, Abcam, Boston, MA, USA), and anti‐phospho‐NF‐κB p65 (Ser536) (1 : 1000; #3033; Cell Signaling Technology). The secondary antibodies used were HRP anti‐rabbit IgG (1 : 5000–1 : 10 000; cat. #7074; Cell Signaling Technology) and horseradish peroxidase (HRP) anti‐mouse IgG (1 : 10 000–1 : 20 000; cat. #7076; Cell Signaling Technology). GAPDH or α‐tubulin was used as internal control to equalize protein loading. The band signal intensity was quantified using imagej software and normalized to that of the control [53].

### Senescence assay

The senescence assay was performed by seeding 3 × 10^4^ cells in a 6‐well culture plate. After 48 h of culture, the cells were transfected with siRNA against *MGB1* or negative control, and then further incubated for 48 h. Then transfection media was discarded and replaced by complete medium for an additional 4 and 5‐days period. Senescent cells were detected using a senescence β‐galactosidase staining kit (Cell Signaling Technology, Tokyo, Japan) according to the manufacturer's protocol. To evaluate the senescence phenotype, the cells were observed under a phase‐contrast microscope (TS100; Nikon Instech Co., Tokyo, Japan) with a 10× objective lens.

### Cell death assay

Dead cells were determined using a trypan blue staining assay, where blue‐stained cells are considered nonviable, whereas the unstained cells are viable. Wild‐type and resistant cells were seeded at a density of 1 × 10^5^ cells per well in a 6 well‐plate for 48 h. The cells were then treated with siRNA against *MGB1* or negative control for 48 h. After 48 h, the cells were collected, stained with trypan blue, and counted manually using Neubauer hemocytometer chamber, then cells were observed under a phase‐contrast microscope (TS100; Nikon Instech Co.) with a 10× objective lens.

### Trastuzumab‐binding assay

The trastuzumab‐binding assay was performed as previously reported but with slight modification [54]. Briefly, each group of cells was seeded at a cell density of 1 × 10^5^ cells per well in a 6‐well plate for 48 h. Cells were then treated with 10 μg·mL^−1^ trastuzumab for 1 h on ice at 4 °C. After cold PBS washing (3 times), a secondary antibody, goat anti‐human IgG H&L conjugated to FITC (1 : 100, cat. ab6854; Abcam), was added to the cells, and was incubated under agitation for 45 min at 4 °C. After cold PBS washing (3 times), fluorescence intensity was measured using Fluoroskan Ascent™ (Thermo Scientific, Thermo Fisher Corporation).

### Generation of MGB1‐overexpressing wild‐type cells

First, we designed and generated an expression vector encoding MGB1 protein, and its sequence was obtained by the following means. RNA was extracted from SK‐BR‐3‐resistant cells. A complementary DNA (cDNA) pool was obtained by RT‐PCR using a ReverTra AceH qPCR RT kit (Toyobo). *MGB1*‐encoding DNA was then amplified from this cDNA sequence by PCR using the KOD‐Neo kit (Toyobo). The primer set used for the PCR was as follows: 5′‐CTCAAGCTTCGAATTATGAAGTTGCTGATGGTCCTC‐3′ (forward) and 5′‐ GGAGAGGGGCGGATCTTAAAATAAATCACAAAGACTGCTG‐3′ (reverse). PCR products were purified using a NucleoSpin® gel and a PCR Clean Up kit (Takara Bio, Osaka, Japan). The pIRES2‐ZsGreen1 vector (Clontech Laboratories Inc., Mountain View, CA, USA) was the vector used for *MGB1* overexpression. Subsequently, it was linearized by digestion with EcoR1 and BamHI and purified by electrophoresis. The linearized vector and DNA obtained from the PCR were ligated using the InFusion®HD Cloning kit (Takara Bio). After cloning the *MGB1* sequence into the expression vector, we performed DNA sequencing to check the plasmid sequence. The final construct was introduced into competent *Escherichia coli* cells (HST08 Premium; TaKaRa Bio) and subsequently purified. SK‐BR‐3 wild‐type cells were transfected with the *MGB1*‐overexpressing construct (MGB1ox) using Lipofectamine 3000 (Invitrogen). Transfected cells were selected by culturing them in the presence of 1.5 mg·mL^−1^ G418 (Promega, Madison, WI, USA) for 7 days. Cell populations containing approximately 70% fluorescent protein‐positive cells were used for subsequent experiments.

### Statistical analysis

We used the *P*‐value or confidence interval of 95% (CI) to calculate the statistical significance of our experimental data. The normal distribution of the data was analyzed using the Kolmogorov–Smirnov test (*P* > 0.05, indicating that the data met the normal distribution). In the case of normal data, we determined whether the variance of the two datasets was significantly different using the *F*‐test (*P* < 0.05, indicating that the variance was significantly different). Based on *F*‐test results, we used the Welch's *t*‐test if the data had statistically different variances or the Student's *t*‐test if the data had variances without statistical difference. A 95% CI was also used when the data were compared to the control group. First, the mean of each dataset was calculated. Then the 95% CI was calculated using Microsoft Excel. The two groups were considered to be significantly different if the means ± 95% CI do not overlap the control value [55].

## Conflict of interest

The authors declare no conflict of interest.

## Author contributions

RK, YK, SI, and HH conceived and designed the experiments. RK, YK, and SI prepared the material of experiments. AE, TM, and MY provided cell lines. RK performed experiments. RK, YK, and SI interpreted and analyzed the data. RK, YK, SI and HH wrote the paper.

## Data Availability

All data generated or analyzed during this study are included in this published article.
